# The pH stability of foot-and-mouth disease virus

**DOI:** 10.1186/s12985-017-0897-z

**Published:** 2017-11-28

**Authors:** Hong Yuan, Pinghua Li, Xueqing Ma, Zengjun Lu, Pu Sun, Xingwen Bai, Jing Zhang, Huifang Bao, Yimei Cao, Dong Li, Yuanfang Fu, Yingli Chen, Qifeng Bai, Jie Zhang, Zaixin Liu

**Affiliations:** 1 State Key Laboratory of Veterinary Etiological Biology, OIE/China Foot-and-Mouth Disease Reference Laboratory, Lanzhou Veterinary Research Institute, Chinese Academy of Agricultural Sciences, No. 1 Xujiaping, Yanchangbao, Lanzhou, Gansu 730046 People’s Republic of China; 2 Key Laboratory of Preclinical Study for New Drugs of Gansu Province, School of Basic Medical Sciences, Lanzhou University, Lanzhou, Gansu 730046 People’s Republic of China

**Keywords:** FMDV, pH stability, Substitutions, Uncoating

## Abstract

**ᅟ:**

This review summarized the molecular determinants of the acid stability of FMDV in order to explore the uncoating mechanism of FMDV and improve the acid stability of vaccines.

**Background:**

The foot-and-mouth disease virus (FMDV) capsid is highly acid labile and tends to dissociate into pentameric subunits at acidic condition to release viral RNA for initiating virus replication. However, the acid stability of virus capsid is greatly required for the maintenance of intact virion during the process of virus culture and vaccine production. The conflict between the acid lability in vivo and acid stability in vitro of FMDV capsid promotes the selection of a series of amino acid substitutions which can confer resistance to acid-induced FMDV inactivation. In order to explore the uncoating activity of FMDV and enhance the acid stability of vaccines, we summarized the available works about the pH stability of FMDV.

**Main body of the abstract:**

In this review, we analyzed the intrinsic reasons for the acid instability of FMDV from the structural and functional aspects. We also listed all substitutions obtained by different research methods and showed them in the partial capsid of FMDV. We found that a quadrangle region in the viral capsid was the place where a great many pH-sensitive residues were distributed. As the uncoating event of FMDV is dependent on the pH-sensitive amino acid residues in the capsid, this most pH-sensitive position indicates a potential candidate location for RNA delivery triggered by the acid-induced coat disassociation.

**Short conclusion:**

This review provided an overview of the pH stability of FMDV. The study of pH stability of FMDV not only contributes to the exploration of molecule and mechanism information for FMDV uncoating, but also enlightens the development of FMDV vaccines, including the traditionally inactivated vaccines and the new VLP (virus-like particle) vaccines.

## Background

Foot-and-mouth disease virus (FMDV), the type species of the *Aphthovirus* genus within family *Picornaviridae* [1, 2], is the pathogen of a highly contagious vesicular disease of cloven-hoofed animals [3, 4]. The virion consists of single-stranded positive-sense RNA genome of approximately 8500 nucleotides in length and icosahedron capsid protein [5, 6]. As FMDV contains no envelope, it is relatively sensitive to the stimulation of physical or chemical materials [7].

The generation of an infectious FMDV particle is a complex feat of engineering mainly involved in the process of capsid assembly, RNA encapsidation, and the viral maturation which are regulated by complicated events in infected cells. The virus capsid is assembled by 60 copies of each of four structure proteins (VP1-4) in a stepwise process [8]. Firstly, one copy of protein VP0 (the precursor of VP4 and VP2), VP3, and VP1 folds into a protomer, then five copies of protomer compose a pentamer, lastly, 12 pentamers assemble into an oligomeric protein shell [7, 9]. There are, at present, two possibilities about how the RNA is encapsidated. One is that the RNA is packaged into the immature capsid after the arrangement of 12 copies of pentamer into a icosahedral capsid. Alternatively, the other is that the assembly of pentamer and encapsidation of RNA are performed at the same time [10]. Maybe the interaction mechanism between RNA molecule and viral capsid would help us solve this puzzle. The last key procedure of viral maturation is the proteolysis of VP0, contributing to the generation of mature, metastable, and infectious FMDV [11]. Compared to the production of FMDV, the breakdown of FMDV in vivo easily happens at pH slightly below neutrality. The current model of dissociation of FMDV supports the assumption that the acidification system in endosome triggers the direct dissociation of FMDV into pentameric subunits, protein VP4, and RNA without intermediate.

FMDV, like other members of the picornaviridae, has a relatively short infectious cycle in cultured cells, including the adsorption, cell entry, uncoating, viral translation, genome transcription and replication, encapsidation, and maturation, in which uncoating, encapsidation, and maturation are not well defined [2]. The uncoating of FMDV depends on irreversible structural rearrangements triggered by the interaction between the pH-sensitive amino acid residues with other residues in the capsid. Since the high-resolution atomic structure information of FMDV uncoating intermediate has not been determined yet, little is understood about the detailed mechanism for FMDV uncoating. However, the study of the pH stability of FMDV possibly provides clues towards capsid disassembly of FMDV from non-structural perspective. With the increasing works about the pH stability of FMDV available, the exposed features are partly concluded as followings: (i) Several methods are applied to select FMDV variants with different sensitivities in a series of pH buffers. (ii) A single amino acid substitution in the capsid of FMDV can increase acid stability and instability [12–16]. (iii) Amino acid residue in different locations of capsid has different degrees of resistance to acid-induced disassembly [12, 14, 15]. (iv) A great many amino acid residues responsible for the acid sensitivity of FMDV have been discovered, some of which are carried by different serotype FMDVs, for example, VP1 N17D [14, 17, 18]. (v) A number of amino acid substitutions closely locate in some regions of the capsid, such as, the N terminal of VP1 protein or close to the pentameric interface [16].

FMDV, a well-learnt virus about structure and function, is the most acid-instable virus among the picornaviruses. The significances for the research of the acid stability of FMDV include the followings: (i) The study of amino acid residues which determine the acid sensitivity of FMDV capsid could serve as an approach to explore the structural bases for FMDV capsid dissociation, capsid assembly, and RNA encapsidation. (ii) Some amino acid residues associated with viral resistance to acid inactivation could be introduced into FMDV genome to obtain acid-resistant FMDV mutants, contributing to the improvement of acid stability of inactivated FMD vaccines. (iii) The stability of recombinant empty capsid expressed in acidic insect cells could be enhanced by introducing the acid-stable residues, leading to an increase in the efficacy of empty capsid vaccine preparations. (iv) The acid-stable virus particles, which are also considered as nanoscale material, could not only act as a vector delivering drugs and genes to special position, but also enlighten the design and manufacture of non-viral nanoparticles. (v) The detailed molecular mechanisms of capsid dissociation benefit the design of antiviral drugs which promote the disassembly of virus shell into subunits. Therefore, this review specifically discussed the recent advances and progresses on the pH stability of FMDV, hoping to better understand the uncoating mechanism of FMDV and provide some inspirations for the virologists, structuralists, and biochemists.

## Review

### The intrinsic reasons for the acid instability of FMDV

#### Acid sensitivity in view of FMDV structure

Non-enveloped FMDV is a spherical and smooth virion without the feature of “canyon” or “pit” structure on the coat [10]. Capsid and RNA element are the determinants of acid sensitivity of FMDV, especially the capsid. The pseudo *T* = 3 icosahedral capsid composed by protein VP1-VP4 can protect RNA from degradation and induce host cell to produce protective immune response. The core structure of protein VP1-VP3 is broadly similar, which contains eight β-barrel folds named alphabetically B to I. Moreover, VP1, VP2, and VP3 are interactively connected by many loops inside or outside capsid, showing that their N-terminus are always located in the inner face and C-terminus are situated in the outer surface [10, 19]. In the case of FMDV, VP1 is smaller than the corresponding protein of other picornaviruses. Part of β-barrel folds of VP1 distributes around the five-fold axes, delimiting the periphery of pore at the five-fold axes of symmetry. The C-terminus of VP1 traverses the external of virion to make the capsid flat. The neighboring N-terminus of VP2 surrounds the three-fold axes of symmetry, forming a tight C structure which may be the calcium-binding site and plays a considerably vital function on keeping capsid stable. Protein VP3 arranged around the three-fold axes is crucial for the capsid stability. Its N-terminus joints together to build a β-annulus, generating a channel around the five-fold axes [10]. Protein VP4, a small, highly hydrophobic protein located in the interior of capsid with a myristoylated N-terminal ends, has been supposed to facilitate the formation of the ion channel and membrane permeability of endosome. The N terminus of VP4 is close to the five-fold axes and the C-terminus is adjacent to the three-fold axes [7, 10]. As referred to the RNA molecule of FMDV, it has been investigated that the RNA genome is more than the genetic material of FMDV, but also involved in the destabilization of virus capsids. One research reports that empty capsid is more pH stable than the homologous virus by 0.5 pH [20]. Some studies also indicate that RNA molecule has the ability to influence the assembly of capsid protein and the capsid protein could assist RNA molecule to fold into a relatively loose and flexible structure [21, 22].

The assembly and stability of viral capsid depend on establishment of a variety of interactions between the capsid subunits and nucleic acid, including hydrophobic interactions, hydrogen bonds, salt bridges, van der Waals forces, covalent bonds, electrovalent bonds, disulfide bonds [23–25]. However, those electrostatic interactions are easily affected when the charged status of amino acid residues and RNA are slightly altered along with the changing pH in the environment. One of the key factors regulating capsid stability-instability balance is the interactions nearby the interpentameric interface which is formed by two neighboring protomers and related to the residues in VP2 and VP3 proteins close to the two-fold axis of icosahedron [7, 23, 26, 27]. It has been proved that altogether 61 residues in a protomer dispersing at interpentameric interface participate in the direct interpentameric interaction, of which only 42 residues have the ability to form noncovalent interactions. Furthermore, truncation of side chains forming buried salt bridges causes genotypic reversion, and truncation of side chains forming buried hydrogen bonds leads to the largely decrease in viral titer [24]. Some other reports demonstrate that a cluster of histidine residues is another significant inducement of the instability of interpentameric interface for the approximation between the pKa of histidine and the pH value at which FMDV disassembles. Seven histidine residues (H21, H65, H87, and H157 in VP2, and H141, H144, and H191 in VP3) are closely located at interpentameric interface according to the 3D structure of O serotype FMDV. They could establish effective electrostatic interactions with charged residues in adjacent pentamers. However, the amount of positive charges only around residue VP3H141 and VP3H144 is much more than that of negative charges. The enhancement of electrostatic repulsions between these two protonated histidine residues and nearby protonated His, Arg, and Lys residues in acid surroundings would greatly weaken the capsid stability [13, 28–30].

#### FMDV uncoating triggered by acid instability

Virus uncoating is a stepwise procedure spatially and temporally controlled within cells, and two committed events should be concerned: (i) The conformation alteration and exhibition of internal proteins to the surface; (ii) Avoidance of premature exposure of virus RNA which could be identified by the sensors of intracellular defense system and evasion of uncovered RNA from harmful substances in the endocytic vesicle [31]. Uncoating program is linked to the breakage of interactions between capsid subunits by diverse host factors. Thus, the viruses employ a great many methods to guide the uncoating, for example, the conformational arrangements induced by the cell receptors, the proteases, chemical elements including low endosome pH and oxidoreductases, and mechanical forces offered by molecular motor [31].

FMDV particles display high sensitivity to acidic environment and easily disassemble into pentameric subunits at a pH close to neutrality [12, 14–16, 20, 32]. In vivo, capsid disassembly of FMDV is related to the viral uncoating, while the FMDV uncoating is triggered by the acidification in the early endosome of host cell. With the pH in the early endosome continually dropping due to the pumping of H+ in the endocytic vesicle, the pH sensors in virus particles are activated and the viral uncoating is triggered [33–35]. The pH sensors detecting subtle variation of pH value are lots of special amino acid residues which change their charged states closely correlative with the pH in the endosome compartment. The pKa of Histidine is 6.4 and the imidazole ring of it could be positively charged in the pH scope of early endosome, all laying the foundation for being an important sensor [13, 29, 36–38]. Owing to the gradual protonation of a wide variety of pH sensors in the capsid, the electrostatic repulsions between different protein subunits in the capsid surface strengthen and the acid instability of FMDV capsid increase. As a consequence, a few internal proteins and active sites in FMDV are exposed, which facilitate the release of RNA genome into the cytosol [10, 39, 40]. Even so, in contrast to the uncoating research of other picornaviruses, the uncoating mechanism of FMDV remains poorly understood.

#### The conflict between acid instability in vivo and acid stability in vitro of the FMDV capsid

The unique capsid construction of FMDV is a result of progressive evolution for the best survival in nature. In vivo, the FMDV capsid is flexible and metastable for the conformational transformation and function implement [23]. But, in vitro, the intact protein shell has a limited ability to deal with the damage of toxic substances [41]. In practice, plenty of factors in the production, storage, and application will influence the pH in the medium and vaccine products, resulting in the degradation of integrated virus particles, damage of antigenic structure, and reduction of immunogenicity. From the above, the capsid of FMDV should hold a delicate balance between acid instability to achieve rapid proliferation in infected cells and the acid stability to resist the aggression in the external environment [42]. This equilibrium is also extraordinarily important for the production of VLPs. One work reporting the introduction of some acid-stable amino acid residues into the empty capsid-like particles suggests that acid-resistant transformation could improve the stability of assembled capsid expressed in inset cells, but the expression yield is seriously suppressed [43].

Interestingly, the compensation effects restoring viability are occasionally detected in the researche of acid sensitivity of FMDV [12, 14, 16, 18, 44]. The FMDV C-S8c1 with substitution VP3 A118V exhibits similar acid stability to the variant containing double mutations VP3 A118V/VP1 N47D, whereas the virus with single mutation displays smaller plaque phenotype and lower titer than the one with double mutations, demonstrating that the substitution VP1 N47D could compensate the negative impact of the substitution VP3 A118V on the virus growth [12]. Another substitution of VP3 D9V located in the outside surface close to the holes around five-fold axes is observed to accompany the mutation VP1 N17D in the same virus, and is speculated to recover the impaired biological characteristics caused by substitution VP1 N17D [14]. While the compensatory substitutions arising frequently at interpentameric and/or nearby the interpentameric interface during virus evolution enable the toleration to the deleterious effects on the virus replication, infectivity, and virulence exerted by the mutations which alter capsid stability [24, 45], it may be served as a strategy to alleviate the conflict between the increase of pH stability and the decrease of fitness.

### The methods to study the influence of pH on FMDV stability

#### The selection of amino acid residues responsible for the acid stability phenotype of FMDV

Asingle amino acid residue substitution in the capsid of FMDV is found to be sufficient to resist the acid dissociation, but its ability is greatly different according to the FMDV serotype, strain, and position in the capsid. Since the more acid-stable FMDV in nature is not easy to be separated, the isolation of virion with acid stability principally depends on the artificial selection. To date, the popular method used to study acid stability of FMDV is that the mutants with increased acid resistance are isolated by several cycles of acid treatment and serially passages. Then, the complete capsid coding regions of the mutants and the parental virus are sequenced and compared to identify the genotypic changes responsible for the acid sensitivity of FMDV. Finally, the recombinant FMDV containing the amino acid replacement found in the acid-resistant FMDV is rescued by using reverse genetics technology. The judgement on whether the selected amino acid residues are responsible for the acid resistance of FMDV is based on the analysis of remaining infectivity of recombinant virus in PBS solutions of different pHs [14, 15, 18, 44]. However, the increased acid stability tends to restrain the endosome acidification of virus, leading to the reduced fitness of FMDV.

#### The selection of amino acid residues with acid-labile ability

There are some researche on the relationship between the increase in acid resistance and the sensitivity to drugs that change the pH in the endosome. According to those researche, the method of endosome acidification blockage is used to select the FMDV mutants with acid instability [12, 16]. Some drugs, such as weak base NH_4_Cl, bafilomycin, concanamycin, and monesin, could weaken endosome acidification by preventing vacuolar ATPase or neutralizing endosome pH using protonophore, causing the impairment of membrane fusion or membrane permeability [46–48]. NH_4_Cl availably raising the pH in the endosome is frequently employed to select FMDV with enhanced acid instability. This drug distinctly influences the early steps of FMDV infection without interfering in the binding of FMDV with cell receptors or internalization pathway. The selection process is as follows: FMDV is continuously inoculated in the BHK-21 cells treated by the NH_4_Cl in advance, then the mutant FMDVs with improved acid sensitivity are obtained and the amino acid residues responsible for the acid lability are discovered [12, 16]. This selection approach is an opposite strategy compared to the first method above. It is reported that the selection frequency of FMDV mutants with acid instability by NH_4_Cl treatment is 10^-1^, while the selection frequency of acid-resistant FMDV mutants is 2.9 × 10^-5^, suggesting that endosome acidification blockage using NH_4_Cl could largely select amino acid residues increasing the acid lability of FMDV. The higher selection frequency with NH_4_Cl will be helpful to locate the regions where plenty of acid-labile residues distribute and provide molecular mechanism for uncoating [12, 49].

#### The chemical calculation to study the pH stability of FMDV

Nearly 20 years ago, the pH stability research of FMDV capsid was conducted from the perspective of chemical calculations [29]. The finite difference Poisson-Boltzmann method which is utilized to compute the macromolecular electrostatic free energy is the main principle to calculate the protein pKa and predict the influence of pH on the capsid stability [50–54]. At first, the titration curves of separate protomer and dimer at different pH values are determined. Secondly, the free energy difference of corresponding values in the two curves is calculated. Eventually, the average charge difference is obtained to confirm the relative pH stability. In their research, the residues within 15 Å of the interface are showed to determine the pH sensitivity of capsid subunits, particularly the two residues VP3 H142 and VP3 H145. However, this method is simply involved in the influence of the titratable residues on the capsid stability, such as Asp, Glu, and His, and fails to analyze the effect of other residues which could be not titrated [29].

### The progresses of the pH stability of FMDV

In fact, FMDV populations are made of a swarm of genetic and phenotypic variants, which are termed as quasispecies [55, 56]. The continuous enhancement of acid stability is not the general feature of FMDV quasispecies due to the negative effect of stronger pH stability on biological functions. In recent years, some FMDVs with different acid-resistant phenotypes are isolated by the artificial selection, which facilitates the identification of molecular basis altering the pH stability of FMDV and provides some guide for the design of engineering virus with pH stability (Table 1).

**Table 1:** The reported amino acid substitutions in the coding region of FMDV capsid

FMDV	Amino acid substitutions				Reference
	VP4	VP2	VP3	VP1	
A12, A119		E131K^b^, D133S^b^		A3S^b^	[28]
A_10_61			H142R^a^, H142F^a^, H142D^a^		[13]
A IND 40/2000			H142R^b^, H142F^b^, H142A^b^, H142D^a^		[30]
Asia1/YS/CHA/05		H145Y ^a^, G192D^b^	K153E^b^	N17D ^a^	[18]
Asia1/JS/05			H140L^a^, H143L^a^		[43]
SAT2, SAT3			H145^b^		[57]
O_1_BFS, A10_61_, A22 Iraq			H142^a^, H145^a^		[29]
O/YS/CHA/05 9A	S73 N^b^	D86A^b^		N17D ^a^	[17]
C-S8c1		F34 L^b^	D9V^b^	N17D ^a^	[14]
C-S8c1		H145Y^a^		N17D ^a^	[15]
C-S8c1		D106G^b^	A123T^b^, A118V^a^		[12]
C-S8c1		G193C^b^	D115E^b^, A116T^a^, A118V^a^, A116V^a^	V11I^a^, T12A^a^, T12I^b^, D17G^b^, Y18H^b^, T22 N^a^	[16]
O1K			T156A^a^	T12N^a^, T2A^a^	[16]

^a^The proved amino acid residues with pH stability or lability

^b^The selected ones which are not found to be responsible for acid sensitivity of FMDV

In 1995, the mutant of FMDV A12 with three mutant residues VP1 A3S, VP2 E131K, and VP2 D133S was isolated. It was found that there were phenotype differences between the mutant virus and parental virus, such as the plaque phenotype, viral infectivity titer, end point in mice, diameter and buoyant density in CsCl, as well as the protein bands in isoelectric focusing [28]. At the same year, the RNA of FMDV was proved to modulate the pH sensitivity of virion [20].

Three years later, the pH-stable curves and chemical calculations were performed for FMDV strains O_1_BFS, A10_61_, and A22 Iraq, indicating that histidine residues VP3 H142 and VP3 H145 greatly influenced the pH-dependent capsid stability [29]. In order to test the theory that histidine-α-helix charge-dipole interaction played an important role in the acid-induced dissociation, the residue VP3 H142 was respectively replaced by Arg, Phe, and Asp in the vaccinia virus expression system. The results revealed that the capsid assembly in the Arg mutant was greatly reduced, while the Phe mutant and Asp mutant were more stable than the wild virus under acidic pH [13]. Lately, four substitutions, VP3 H142R, VP3 H142F, VP3 H142A, and VP3 H142D, were also introduced into the full-length cDNA clone of type A FMDV to rescue FMDV mutants, but only mutant virus including substitutionVP3 H142D was recovered and possessed the increased ability to resist the acid inactivation. In addition, the substitution VP3 H142D did not have any significant impact on the antigenicity of mutant virus as compared to the wild-type virus [30]. Cao et al. reported that the empty capsid-like particles possessing replacements VP3 H140L and VP3 H143L, which were expressed in baculoviruses express system, were more stable at pH blow 7.0 than the wild one [43]. Another research based on the pKa predictions indicated that VP3 H145 could alter the pH stability of SAT2 and SAT3 FMDVs because of the establishment of some interactions with other residues on the pentameric interface [57].

When FMDV C-S8c1 was treated with PBS at pH 6.0 for 30 min, six FMDV mutants with increased acid resistance were acquired. The genetic analysis showed that those variants all carried substitution VP1 N17D, but only some variants contained replacements VP3 D9V and VP2 F34 L. The rescued FMDV possessing substitution VP1 N17D could really resist the acid dissociation through a series of verifications [14]. As this work continued, another mutant FMDV bearing double substitutions VP2 H145Y and VP1 N17D was isolated. It had higher acid resistance and was known as the most acid stable FMDV with the pH_50_ value (a pH value causes a loss of 50% of infectivity) of 5.4 [15]. In addition, the presence of replacements VP2 H145Y and VP1 N17D did not compromise the immunological potential, including the ability to elicit neutralizing antibodies [58]. Later, type O FMDV mutants with acid stability were selected by acid treatment. This virus contained substitutions VP1 N17D, VP2 D86A, and VP4 S73 N. But only the replacement VP1N17D was actually the molecular determinant for the increased acid stability phenotype [44]. Acid-stable Asia1 FMDV mutants containing mutationsVP1N17D, VP2 H145Y, VP2 G192D, and VP3 K153E, were selected too. However, only the replacement VP1N17D or VP2 H145Y could confer acid-resistant ability to FMDV, respectively [18].

In 2010, inhibition of endosomal acidification by NH_4_Cl or concanamycin A was used to isolate acid-labile FMDV mutants. Capsid sequencing of those mutants showed that the substitutions VP3 A123T, VP3 A118V, and VP2 D106G were carried by three C-S8c1 mutants which displayed acid lability. Only the substitution VP3 A118V was revealed to not only sufficiently resist NH_4_Cl and concanamycin A treatment, but also enhance sensitivity of FMDV to acid-induced infection inactivation [12]. Five years later, many amino acid residues substitutions increasing acid sensitivity of FMDV were selected through three experiences in a study. One was that vesicular fluid collected from a pig infected with FMDV C-S8c1 was added to BHK-21 cells treated with NH_4_Cl, which resulted in the isolation of replacement VP3A116V. The second assay was that several passages of different C-S8c1 variants in the presence of NH_4_Cl generated abundant amino acid replacements, for instance, VP2 G193C, VP3 D115E, VP3 A116T, VP3 A118V, VP1 V11I, VP1 T12A, VP1 T12I, VP1 D17G, VP2 Y18H, and VP1 T22 N. The last test related to that the isolated O1K FMDV mutants by NH_4_Cl treatment carried mutations VP3 T156A, VP1 T12 N, and VP1 T2A. The careful analysis of those substitutions indicated that two different regions of FMDV capsid, the N-terminus of VP1 or close to the pentameric interface, contributed to modulate viral particle stability [16].

It was reported that the acid stability of FMDV was connected with cellular Rab GTPases which controlled traffic between different endosome populations. The acid-resistant FMDV mutant was less sensitive to the inhibition of Rab5, but more sensitive to the inhibition of Rab7 or Rab11 [40]. Therefore, other FMDV variations with different pH sensitivity could be possibly selected by the treatment of cellular Rab GTPases, such as Rab5, Rab7, and Rab11.

In summary, with the constant increase of pH stability research of FMDV, more and more amino acid residues substitutions which confer to increase the acid stability or instability of FMDV are being gradually found. Residues VP3 H142 and VP3 H145, which are conserved in FMDVs and situated at interpentameric interface, have been frequently studied for a long time. They are always recognized as the hugely instable factors of FMDV capsid at acidic pH values [13, 29, 30, 57]. Amino acid residue VP1 N17 locates at the internal region of capsid close to the interpentameric interfaces. The nearly isosteric and electronegative substitution VP1 N17D involves the conversion from nonpolar amino acid to negatively charged residue and occurs in the type C, Asia1, and O FMDV mutants with increased acid stability. Although this substitution leads to the disappearance of a hydrogen bond formed by the VP1 N17and VP4 G78 in the same protomer, it really could improve the acidic stability and thermal stability of FMDV [14, 18, 44]. VP2H145 is mapped to the internal region of the capsid at the intraprotomeric interface and has been proposed to participate in the cleavage of VP0 in poliovirus. The substitution VP2H145Y enables type Asia1 and C FMDVs resistance against the acid-induced inactivation [15, 18]. The mutation VP3 A118V, which is located close to the residue VP3 H142 and VP3 H145, could regulate the FMDV growth in the BHK-21 cells in the presence of NH_4_Cl. This substitution introducing a bulkier side chain may produce strong electrostatic repulsions with neighboring histidine residues, leading to elevated tendency to disassemble [12, 16].

### The hypothetical mechanism for FMDV uncoating

To date, the fine structure of the “A-particle” that is formed on the process of viral uncoating has been available for enterovirus 71(EV71), poliovirus (PV), human rhinovirus 2 (HRV2), coxsackievirus A 16 (CAV16), human Cardiovirus Saffold Virus 3 (SAFV-3), and equine rhinitis A virus (ERAV), providing us some enlightenment for the study of FMDV uncoating [59–66]. As for the Enterovirus uncoating, the pores around two-fold axes caused by the secondary conformation rearrangement of virus capsid severed as the channel for the release of genomic RNA [59, 60, 62, 63]. The insights into HRV2 end-stage uncoating by the X-ray structure showed that reorganization in the interprotomeric interfaces resulted in the formation of the biggest pores at the two-fold axes which were strongly suggested as routes for the externalization of VP1 N-terminus and the extrusion of the RNA molecule [66, 67]. Similarly, the pores at icosahedral two-fold axes were hypothesized to be channels for the egress of the CAV16 internal proteins and RNA [65]. However, the expanded A particle of SAFV-3 contained pores between the three-fold and five-fold axes that allowed the externalization of VP1 N-termini and of VP4 subunits [68]. The structure of a massively expanded ERAV particle losing the RNA genome and VP4 protein illustrated that the enough large pores on the three-fold axes may be a possible route to allow RNA egress rather than the small holes near the base of a surface depression or a somewhat larger hole at the two-fold axes [69, 70]. To sum up, the several known uncoating events of picornavirus initiate at different positions including the two-fold axes, three-fold axes and the position between the three-fold and five-fold axes, but similarly involve the uncoating intermediate, VP1 N-terminus, protein VP4, and the RNA genome.

**Fig. 1:**
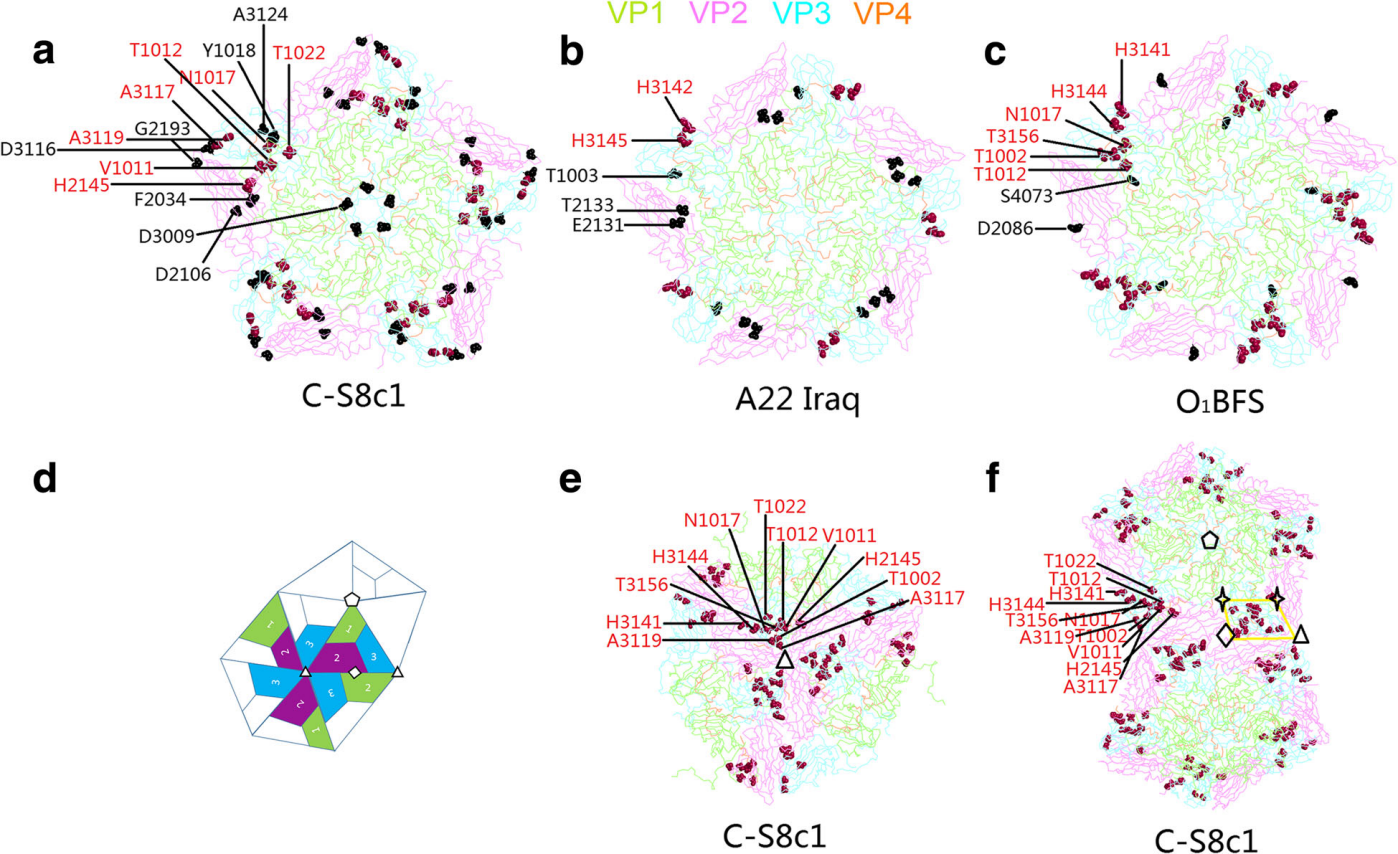
The location in FMDV capsid of amino acid residues found to be substituted in Table 1. **a**, **b** and **c** Outside view of a respective pentamer model of C-S8c1, A22 Iraq and O1BFS (PDB: 1FMD, 4GH4 and 1BBT). The *red* amino acid residues are ones which are marked with ‘a’ in Table 1 and the *black* ones are those residues which are marked with ‘b’ in Table 1. The VP1 is *green*; VP2 is *purple*; VP3 is blue; VP4 is *orange*. According to the gene sequence of FMDV C-S8c1 and A22 Iraq, residues D115, A116, A118 and A123 in Table 1 are labeled as D116, A117, A119 and A124 in panel (**a**), and residues A3 and D133 are labeled as T3 and T133 in panel (**b**). **d** Outside view of schematic structure of part FMDV capsid. The VP1 is green; VP2 is purple; VP3 is blue (five-fold axis, pentagon; three-fold axis, triangle; two-fold axis, diamond). **e** and **f** Outside view of six protomers around a three-fold axis and two pentamers model of C-S8c1 capsid. The color of VP1, VP2, VP3, and VP4 are the same as those in panel **a**, **b**, and **c**. Three-fold axis is triangle; two-fold axis is diamond; five-fold axis is pentagon; intersection of VP1, VP2, and VP3 is four point star. All *red* amino acid residues in panel **a**, **b**, and **c** are displayed in panel **e** and **f**, respectively. Those *red* residues are included in a *yellow* quadrangle region in which the three-fold axis, two-fold axis and the intersections of three capsid proteins (VP1, VP2, and VP3) act as the four vertexes

For FMDV, its uncoating event remains still elusive. The analogous uncoating intermediate product has not been discovered in FMDV, and the priority of genome release and capsid dissociation is not sure as well. Moreover, during the uncoating event, we still have no information on whether the protein VP1, especially the N-terminal residues, isrelated to the conformation rearrangement of the capsid and involve the expelling of FMDV genome. One more attractive question required to be addressed is which site in the capsid is chosen by FMDV for externalization of the viral peptides and viral genome: five-fold axes, two-fold axes, or the three-fold axes? Previous studies on capsid dissociation of FMDV and release of viral genome focused on the regulation of a cluster of histidine amino acid residues lining the pentameric interface. However, recent studies about mutant FMDVs with different levels of acid resistance indicate that many other amino acid residues in the capsid mediate the acid-induced disassembly of FMDV. In this review, we summarize those special residues in Table 1 and display them in the model of different serotype FMDVs. Remarkably, Fig. 1 clearly shows that most of residues listed in the table, particularly those residues which have been identified to be truly responsible for the FMDV dissociation at acid conditions, are mapped in a quadrangle region which involves the three-fold axes, two-fold axes, interprotomer interface, and interpentamer interface. As it is well known that those axes and interfaces play a crucial role in controlling the capsid stability and uncoating event in picornaviruses, the quadrangle region possibly contains the initiation site for FMDV uncoating or at least includes some key amino acid residues which initiate the FMDV uncoating activity.

In FMDV-infected cells, acidification in the endosomes firstly sensed by pH sensors in the FMDV capsid controls the switches of capsid from metastable state to the precise structure rearrangement for initiating the RNA translocation. So, the distribution of those pH sensors could not only imply the location where the FMDV uncoating most possibly initiates, but also provide molecular and mechanistic insights into FMDV genome uncoating. In this review, we conclude that the pH sensors are found to be closely arranged in one region of the capsid. In addition, the evolved interactions which weave the four capsid proteins together are intrinsically weak enough in order to release the genome. In those contexts, even the minor change of the environmental pH gives rise to strong electrical repulsions between the pH sensors in the quadrangle region and their surrounding residues, finally breaking the fragile interactions and facilitating the instantaneous burst of FMDV capsid. Therefore, the uncoating activity of FMDV, in fact, is a dramatic and irreversible conformational change of capsid, which is in line with our common understanding that FMDV is the most pH-unstable picornavirus and uncoating intermediates remain undiscovered.

## Conclusion

In recent years, physics and chemistry are more often recognized as important tools to qualitatively and quantitatively investigate viruses, which boost the research about the effect of pH on the FMDV stability. The delicate capsid structure of FMDV determines the acid lability of virion in favor of the efficient uncoating of FMDV. But the acid stability is also required to keep FMDV integral in vitro. In order to ease this contradiction, a great quantity of FMDV mutants with different pH stability has been isolated by various methods without serious impact on the infection. The FMDV mutants are found to contain many amino acid residue replacements which are partly confirmed to be responsible for the pH-sensitive phonotypes of virus particles. Those substitutions not only provide molecular basis for the uncoating of FMDV which is all not fully understood now, but also could be applied to the medicine and industry for the improvement of FMDV vaccines, antiviral drugs, and nanoscale materials.

In this review, we have compiled the collection of a number of amino acid residues which are susceptible to change their protonation states below pH 7.0 shedding light on the possible positions for the FMDV genome release from the endosome in a view of non-structural study. Here, we propose that the FMDV RNA delivery possibly initiates or involves a quadrangle region in the capsid. In the future, once the high resolution crystal structures of different FMDV uncoating intermediates have been achieved, the pH-triggered structural alterations and specific interactions changes involved in a hinge-type movement and degradation of the capsid would become available in detail. At that time, we would clearly confirm the real strategy for the uncoating event of FMDV.

## Abbreviations

2A^pro^: 2A protease
3C^pro^: 3C protease
CAV-16: Coxsackievirus A 16
ERAV: Equine rhinitis A virus
EV71: Enterovirus 71
FMD: Foot-and-mouth disease
FMDV: Foot-and-mouth disease virus
HRV-2: Human rhinovirus 2
L^pro^: Leader protease
pH_50_ value: The pH causes a loss of 50% of infectivity
PV: Poliovirus
SAFV-3: Human Cardiovirus Saffold Virus 3
VLPs: Virus-like particles
